# Shaking culture attenuates circadian rhythms in induced pluripotent stem cells during osteogenic differentiation through the TEAD-Fbxl3-CRY axis

**DOI:** 10.1038/s41420-025-02533-6

**Published:** 2025-05-24

**Authors:** Yunyu Fu, Hiroko Okawa, Naruephorn Vinaikosol, Satomi Mori, Phoonsuk Limraksasin, Praphawi Nattasit, Yu Tahara, Hiroshi Egusa

**Affiliations:** 1 Division of Molecular and Regenerative Prosthodontics, Tohoku University Graduate School of Dentistry, Sendai, Miyagi Japan; 2 Center of Excellence for Dental Stem Cell Biology, Faculty of Dentistry, Chulalongkorn University, Bangkok, Thailand; 3 Department of Oral Medicine and Periodontology, Faculty of Dentistry, Mahidol University, Bangkok, Thailand; 4 Graduate School of Biomedical and Health Sciences, Hiroshima University, Hiroshima, Japan; 5 Center for Advanced Stem Cell and Regenerative Research, Tohoku University Graduate School of Dentistry, Sendai, Miyagi Japan

**Keywords:** Multipotent stem cells, Stem-cell research

## Abstract

Circadian rhythms, which synchronize cellular and organismal activities with the Earth’s 24-hour light-dark cycle, are controlled by clock genes. These genes not only regulate metabolic and physiological processes but also influence osteogenesis. Despite extensive research on the genetic control of circadian rhythms, little is known about the mechanisms by which mechanical factors in the extracellular environment affect these rhythms during the osteogenic differentiation of induced pluripotent stem cells (iPSCs). Shaking culture, which promotes the formation of three-dimensional organoid-like constructs from iPSC embryoid bodies (iPSC-EBs), introduces distinct biomechanical forces compared with static adherent culture. This raises the question of how these forces affect the circadian gene expression during osteogenic differentiation. In this study, we investigated the effects of shaking cultures on the circadian rhythm of key clock genes (*Clock*, *Bmal1*, and *Npas2*) in iPSC-EBs. In the adherent culture, iPSC-EBs displayed rhythmic oscillations of the clock genes, which were attenuated in the shaking culture. RNA-seq analysis revealed that the yes-associated protein (YAP)-transcriptional enhanced associate domain (TEAD) transcriptional cascade was activated in the shaking culture. Further investigations using assay for transposase-accessible chromatin with sequencing and chromatin immunoprecipitation assays identified Fbxl3 as a direct target of this transcriptional cascade. Fbxl3 upregulation in the shaking culture enhanced the degradation of CRY proteins, which are essential components of the circadian feedback loop, thereby suppressing clock gene oscillations. In addition, treatment with verteporfin, a YAP-TEAD inhibitor, restored circadian gene oscillations and increased the expression of osteogenic markers in shaking culture. These findings highlight a novel mechanistic link between biomechanical cues and circadian regulation and offer potential insights for optimizing tissue engineering strategies in regenerative medicine.

## Introduction

Cells and organisms have evolved an approximately 24-h internal circadian clock in response to environmental cues from the solar cycle [1]. Molecular regulation of the circadian clock involves a transcriptional-translational feedback loop regulated by core clock genes. In this feedback loop, the CLOCK-brain and muscle arnt-like (BMAL1) complex activates the transcription of *Per* and *Cry*, whose proteins inhibit CLOCK/BMAL1 activity and drive the cyclic expression of these circadian genes [2]. Neuronal PAS domain 2 (NPAS2), a paralog of CLOCK, can substitute CLOCK to form heterodimers with BMAL1 [3]. The central circadian clock is located in the suprachiasmatic nucleus and regulates peripheral clocks via neuroendocrine signaling [4, 5]. Mechanical forces from the extracellular environment also influence circadian rhythms [6]. Alterations in extracellular matrix (ECM) biomechanics during aging and tumorigenesis can lead to circadian dysregulation [7, 8]. Additionally, changes in intercellular adhesion and variations in culture density correlate with intracellular circadian rhythms [9]. These mechanical stresses may trigger physiological responses via mechanoreceptors, ultimately affecting the circadian rhythm.

Induced pluripotent stem cells (iPSCs) possess unlimited proliferative potential and pluripotency, making them ideal cell sources for tissue engineering in dental treatments [10, 11]. Additionally, iPSCs can self-organize and spontaneously assemble to form three-dimensional (3D) organoids when cultured on low-adhesion surfaces. We successfully established osteogenic differentiation protocols for mouse iPSCs in adherent (static) [12] and floating (shaking) 3D cultures [13]. Shaking cultures exert significantly different mechanical forces than adherent cultures [14]. Circadian regulation is important in bone biology [15, 16]. Mutations, including deletions, in *Clock*, *Npas2*, *Bmal1*, and *Cry2* greatly affect bone formation [17–21]. However, how these mechanical forces affect circadian rhythms during the osteogenic differentiation of iPSCs is unclear.

In this study, we explored the relationship between circadian rhythms, osteogenic differentiation of iPSCs, and mechanical force in shaking cultures by comparing the molecular profiles of adherent and shaking cultures. We showed that the mechanical forces applied on iPSCs embryoid bodies (iPSC-EBs) in shaking cultures attenuated intracellular rhythmic oscillations through the transcriptional enhanced associate domain (TEAD)-F-box only protein 13 (Fbxl3)-CRY axis. The yes-associated protein (YAP)-TEAD inhibitor restored the circadian oscillations of iPSCs, thereby promoting osteogenesis. This study provides new insights into the optimization of iPSC-based bone-organoid fabrication.

## Results

### Shaking culture attenuated the oscillatory expression of clock genes during osteogenic differentiation

**Fig. 1:**
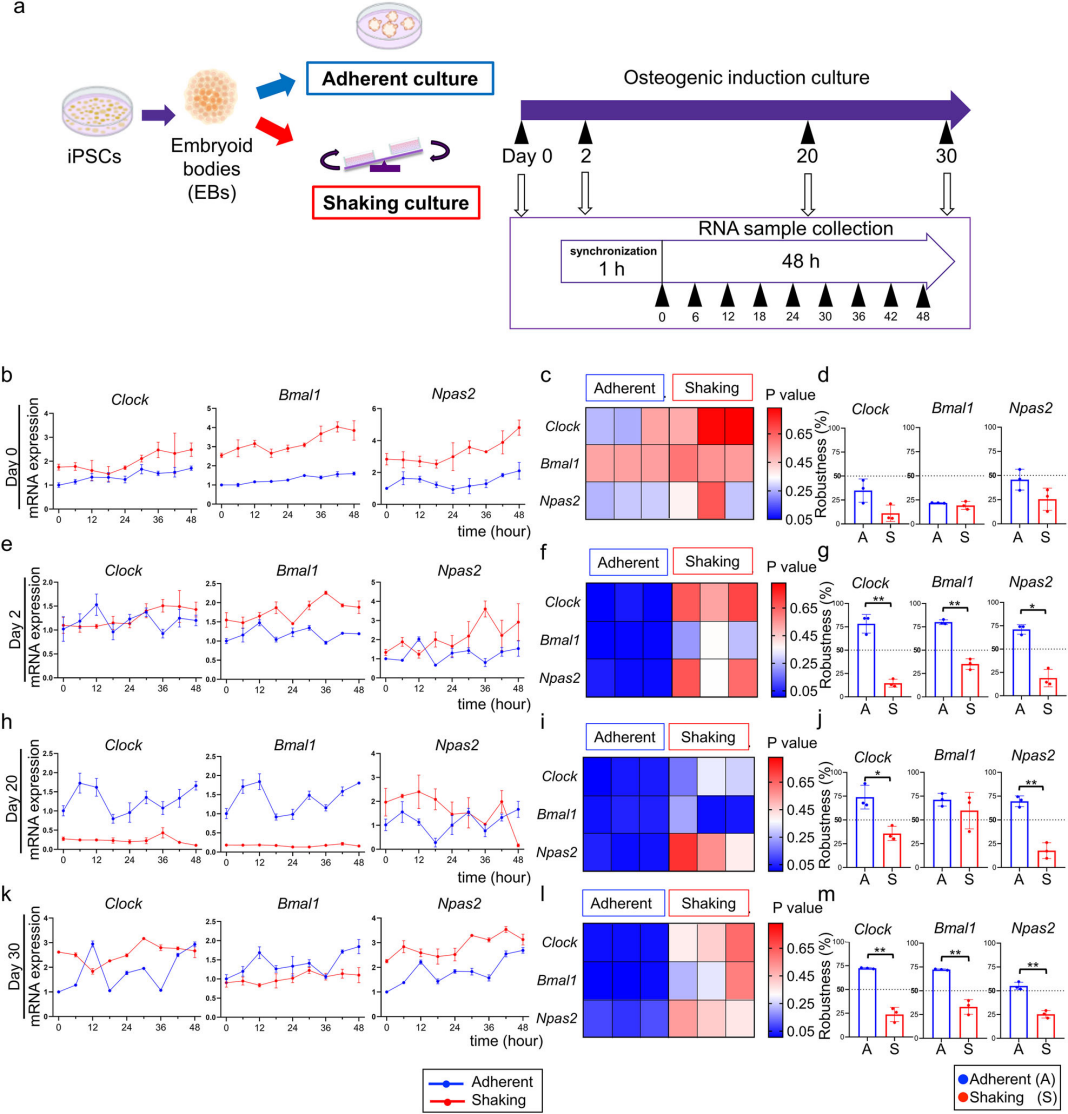
Expression profiles of clock genes in adherent and shaking cultures during osteogenic differentiation. **a** Schematic illustration of the time course for measuring circadian rhythms. RNA samples were collected every 6 h for 48 h after synchronization. Real-time RT-PCR analysis of *Clock*, *Bmal1*, and *Npas2* mRNA levels at the indicated times in iPSC-EBs before differentiation (**b**) and on days 2 (**e**), 20 (**h**), and 30 (**k**). **c**, **f**, **i**, **l** *P* values were obtained using Cosinor software by comparing the real-time RT-PCR curve with the standard cosine curve. Smaller *P* values indicate a better fit of the cosine curve to the PCR results, suggesting a better circadian rhythm. Three grids in each group represent triplicate experiments. **d**, **g**, **j**, **m** Robustness values obtained using Cosinor software. Higher robustness values indicate more regular oscillations in the PCR data, reflecting a stronger rhythmic pattern. *P < 0.05, **P < 0.01 (one-way ANOVA and Tukey’s test; *n* = 3).

We used the shaking culture condition, which was shown to promote the expression of osteogenic genes, to facilitate the formation of 3D bone-like organoids by iPSCs in our previous report [13]. To assess the effect of mechanical forces in the shaking culture on the rhythmic expression of clock genes during osteogenic differentiation, we compared the expression profiles of *Clock, Bmal1*, and *Npas2* in adherent and shaking cultures (Fig. 1a). *P* < 0.05 and a robustness value > 50% indicated a rhythmic oscillation of clock gene expression. No significant oscillations were observed in undifferentiated iPSCs in either culture (*P* > 0.05 and robustness values < 50%; Fig. 1b–d), suggesting that clock genes do not exhibit circadian rhythms before differentiation.

In the shaking culture, clock genes exhibited *P* > 0.05 and robustness values < 50% after 2 d of osteogenic differentiation, indicating persistent disruption of oscillatory expression (Fig. 1e–g). This pattern persisted into the mid-stage of differentiation (day 20), with *Clock*, *Bmal1*, and *Npas*2 still lacking circadian oscillations (Fig. 1h–j). By day 30, although the shaking culture group exhibited slight changes in oscillations, *P* > 0.05 and robustness values < 50% were determined, indicating a continued absence of rhythmicity (Fig. 1k–m). These results suggest that the shaking culture conditions attenuate the oscillatory expression of clock genes.

In contrast, the adherent culture consistently showed *P* < 0.05 and robustness values > 50% from days 2–30 of osteogenic differentiation, indicating rhythmic oscillations in clock gene expression (Fig. 1e–m).

### YAP-TEAD transcriptional cascade as a potential regulator of rhythmic oscillations

**Fig. 2:**
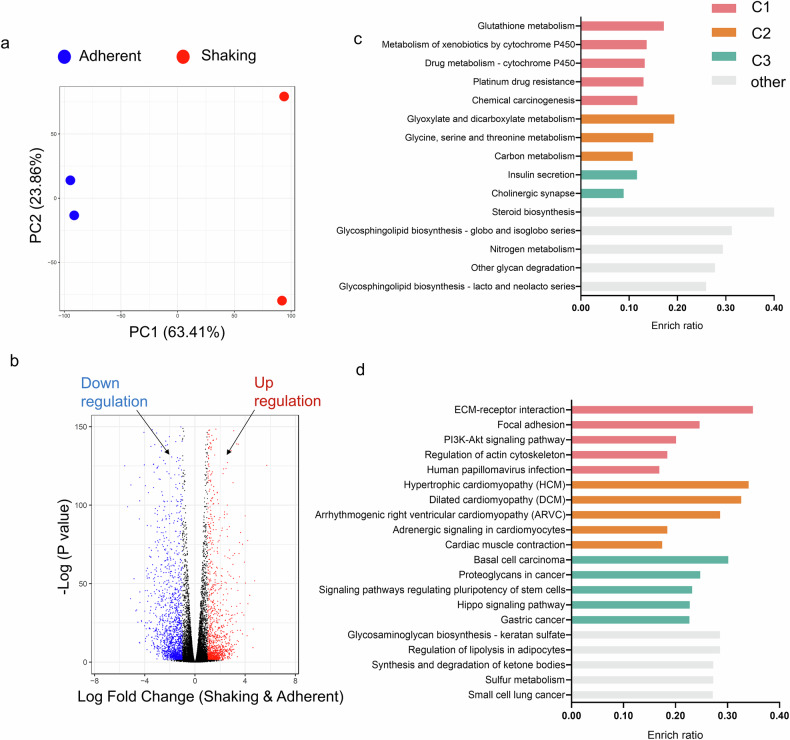
RNA-seq analysis of iPSCs-EBs cultured under adherent and shaking conditions. **a** Multidimensional scaling plot of different culture conditions. **b** Volcano plot of significant DEGs. Red and blue points represent the upregulated and downregulated DEGs, respectively. **c** KEGG enrichment analysis of the upregulated genes in the shaking culture group. **d** KEGG enrichment analysis of the downregulated genes in the shaking culture group.

We investigated the mechanisms underlying the attenuated oscillations using RNA-sequencing (RNA-seq) and assay for transposase-accessible chromatin with sequencing (ATAC-seq). A multidimensional scaling plot revealed a clear distinction between the two culture conditions (Fig. 2a). A total of 1,378 upregulated and 1,774 downregulated genes were identified (Fig. 2b).

The potential signaling pathways involved were identified. In the shaking culture, the top-ranked pathways among the upregulated genes were related to metabolism, including glutathione, glyoxylate, and dicarboxylate metabolism (Fig. 2c). For the downregulated genes, pathways associated with ECM-receptor interaction, focal adhesion, and actin cytoskeleton regulation were enriched in cluster 1, while pathways regulating stem cell pluripotency were enriched in cluster 2. Additionally, the Hippo pathway, which is critical for mechanical signal transduction [22], was downregulated (Fig. 2d). The key effector of this pathway is the YAP-TEAD transcriptional cascade, which has been implicated in circadian rhythm regulation [23].

**Fig. 3:**
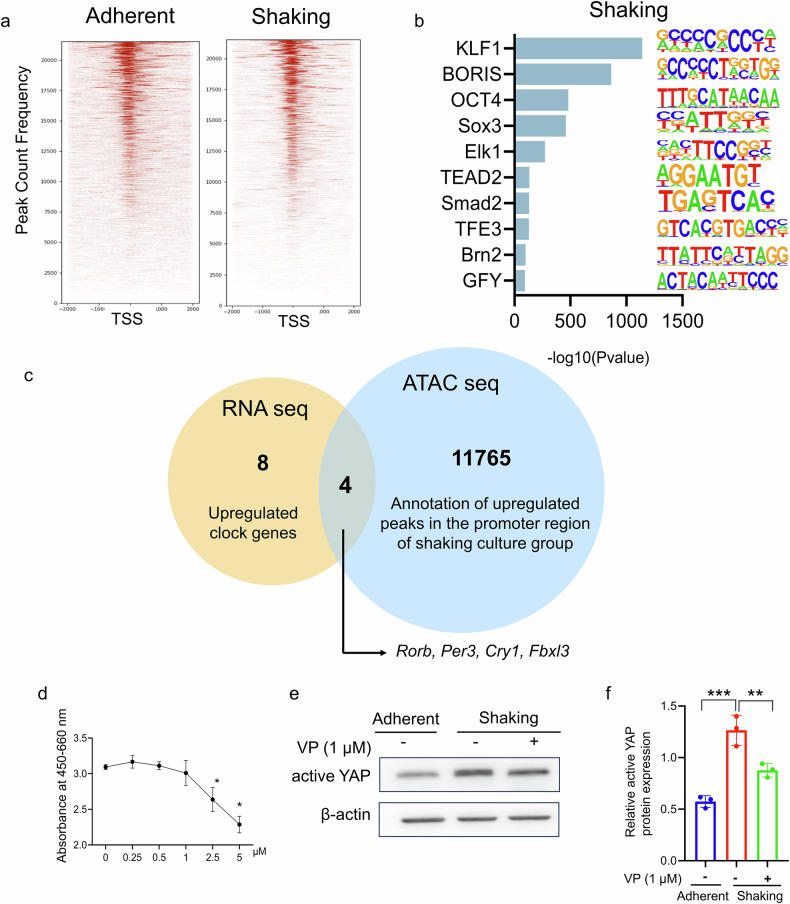
Prediction of potential target genes of TEAD using combined RNA-seq and ATAC-seq. **a** Heatmap analysis of average profile binding in TSS regions. **b** Top 10 motifs enriched in the promoters of upregulated genes in the shaking group. **c** Venn diagram showing the overlap between the upregulated genes identified using RNA-seq and the genes with increased chromatin accessibility of the promoter regions identified using ATAC-seq. The overlap represents the genes present in both datasets. **d** Cytotoxicity assay of iPSC-EBs treated with the YAP-TEAD interaction inhibitor verteporfin (VP) at concentrations ranging from 0 to 5 μM. Asterisks indicate significant differences compared with the untreated group. **e** Western blot analysis of the YAP nuclear translocation marker (active YAP) in the adherent and shaking groups with or without VP treatment. **f** Quantitative analysis of active YAP expression using ImageJ software. Expression levels were normalized to that of β-actin. **P < 0.01, ***P < 0.001 (one-way ANOVA and Tukey’s test; *n* = 3).

We explored the underlying key molecular mediators that attenuate rhythmicity in shaking cultures. Chromatin accessibility was enriched around the transcription start sites (TSSs) (Fig. 3a). Transcription factor-binding site enrichment analysis identified potential transcription factors associated with regions with increased chromatin accessibility. TEAD2 was enriched in regions with accessible chromatin in the shaking group but not in the adherent group (Fig. 3b and Supplementary Fig. 1). These results suggest that the YAP-TEAD transcriptional cascade is a crucial mediator of circadian rhythm attenuation in shaking cultures.

We then searched for the genes regulated by TEAD. The genes activated by YAP-TEAD-mediated transcription were expected to show increased expression and greater promoter region chromatin accessibility in shaking cultures. Analyzing the upregulated circadian rhythm-related genes in the RNA-seq data (Supplementary Table 1) and cross-referencing them with genes showing increased promoter region peaks in the ATAC-seq data led to the identification of the following four candidate genes: *Rorb, Per3, Cry1*, and *Fbxl3* (Fig. 3c). Although Per1, Per2, and Per3 participate in the circadian rhythm feedback loop, targeted Per3 disruption in mice only slightly affects the circadian phenotype [24]. Therefore, we focused on *Rorb*, *Cry1*, and *Fbxl3* in the subsequent experiments.

The YAP-TEAD inhibitor verteporfin (VP) [25] was added to the cultures. A 1 μM concentration was used because higher concentrations significantly increased cytotoxicity (Fig. 3d). Western blot analysis showed that the shaking condition increased nuclear YAP (active YAP), which were attenuated by VP treatment (Fig. 3e, f).

**Fig. 4:**
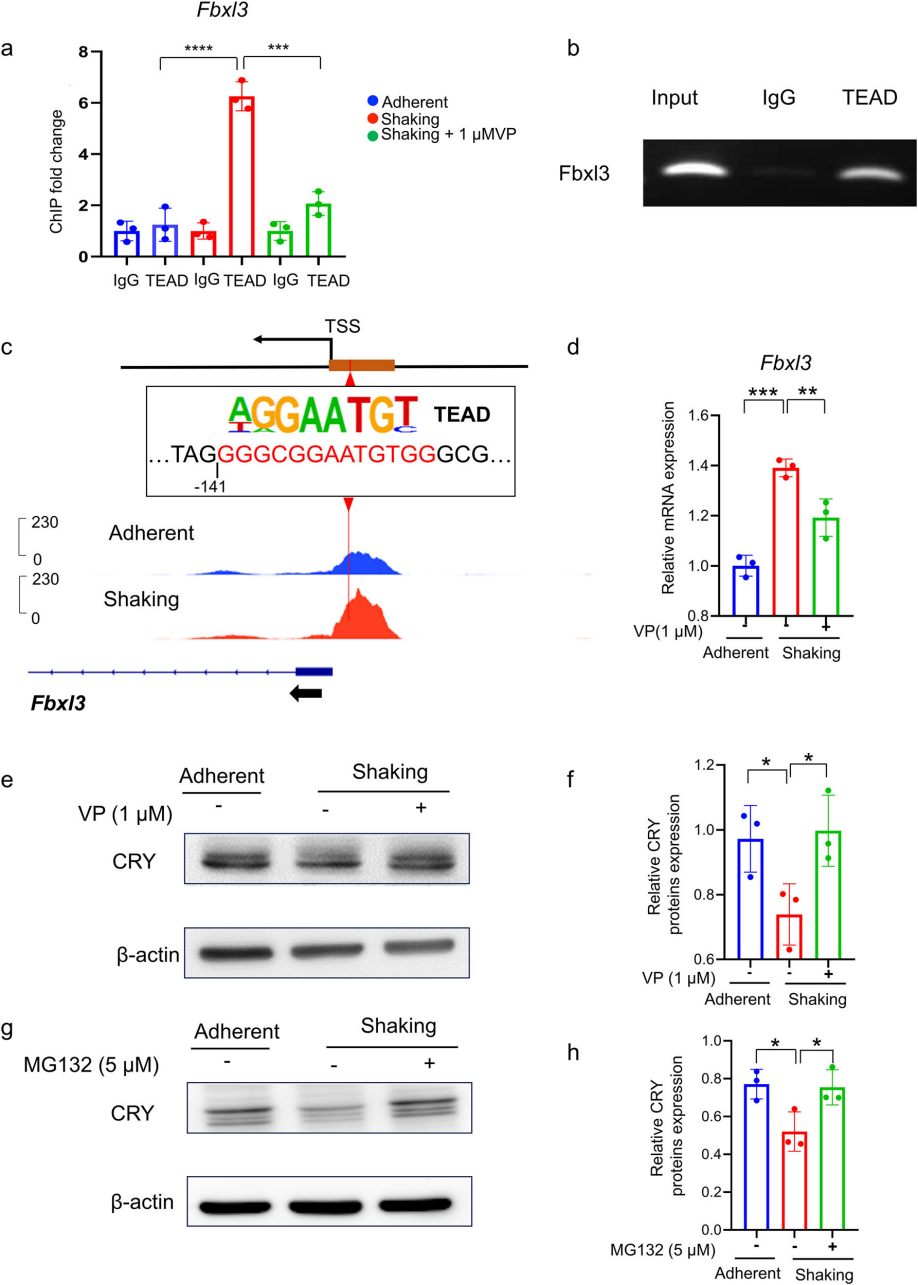
*Fbxl3* is a target gene of TEAD and affects circadian rhythms by regulating the ubiquitination of CRY proteins. **a** ChIP-qPCR analysis of the interaction between TEAD and the *Fbxl3* promoter. The input represents an aliquot of total DNA. **b** ChIP-qPCR analysis of binding between TEAD and the *Fbxl3* promoter under adherent and shaking conditions with or without VP treatment. **c** Schematic representation of the Fbxl3 promoter and predicted TEAD binding site. Predictions were performed using the JASPAR software. Genome browser track of ATAC-seq peaks for Fbxl3 showing higher peaks at promoter regions in the shaking culture group, with the predicted binding site located among them. **d** Real-time RT-PCR analysis of the relative expression level of *Fbxl3*. **e** Western blot analysis of CRY proteins, whose increased degradation in the shaking culture group disrupts the feedback loops of the circadian rhythm. **f** Quantitative analysis of CRY protein expression performed using ImageJ software. Expression levels were normalized to that of β-actin. **g** Western blot analysis of CRY proteins under adherent and shaking conditions with or without 5 μM MG132 treatment. **h** Quantitative analysis of CRY protein expression performed using ImageJ software. Expression levels were normalized to that of β-actin. *P < 0.05, **P < 0.01, ***P < 0.001 (one-way ANOVA and Tukey’s test; *n* = 3).

If the candidate genes are regulated by YAP-TEAD, binding of TEAD to the promoter regions of the genes should be higher in the shaking group than in the adherent group and should be weakened by VP treatment, thereby reducing gene expression. To confirm this, chromatin immunoprecipitation (ChIP) assays were conducted to detect changes in the binding of TEAD to the three candidate molecules. Only Fbxl3 showed a significant increase in TEAD binding under shaking conditions, which was reduced by VP treatment (Fig. 4a Supplementary Fig. 2). ChIP-qPCR confirmed the binding of TEAD to the Fbxl3 promoter (Fig. 4b). The accessible chromatin region in the Fbxl3 promoter exhibited a higher ATAC peak in the shaking group and contained a TEAD-binding site (Fig. 4c). These findings suggest that Fbxl3 is a target of the YAP-TEAD transcriptional cascade, which modulates oscillatory clock gene expression.

Fbxl3 plays a role in circadian rhythm regulation, and its upregulation can result in abnormal CRY protein degradation [26], potentially disrupting the feedback loop of clock gene oscillations. To examine whether the interaction among TEAD, FBXL3, and CRY contributes to circadian rhythm attenuation in shaking cultures, we analyzed Fbxl3 mRNA and CRY protein expression levels. In the shaking group, Fbxl3 was upregulated (Fig. 4d) whereas CRY was downregulated, whereas this phenomenon was reversed by VP treatment (Fig. 4e, f). Because CRY degradation is ubiquitin-mediated, the ubiquitin-proteasome inhibitor MG132 was added to the shaking group. MG132 treatment upregulated the CRY protein in the shaking group by inhibiting ubiquitin-mediated degradation (Fig. 4g, h), implying that CRY protein degradation in this group was mediated by Fbxl3-driven ubiquitination. These findings demonstrate that the TEAD-FBXL3-CRY axis is involved in disrupting the negative circadian feedback loop in shaking cultures.

### Treatment with a YAP-TEAD inhibitor restored the rhythmic expression of clock genes in the shaking culture during osteogenic differentiation of iPSCs

**Fig. 5:**
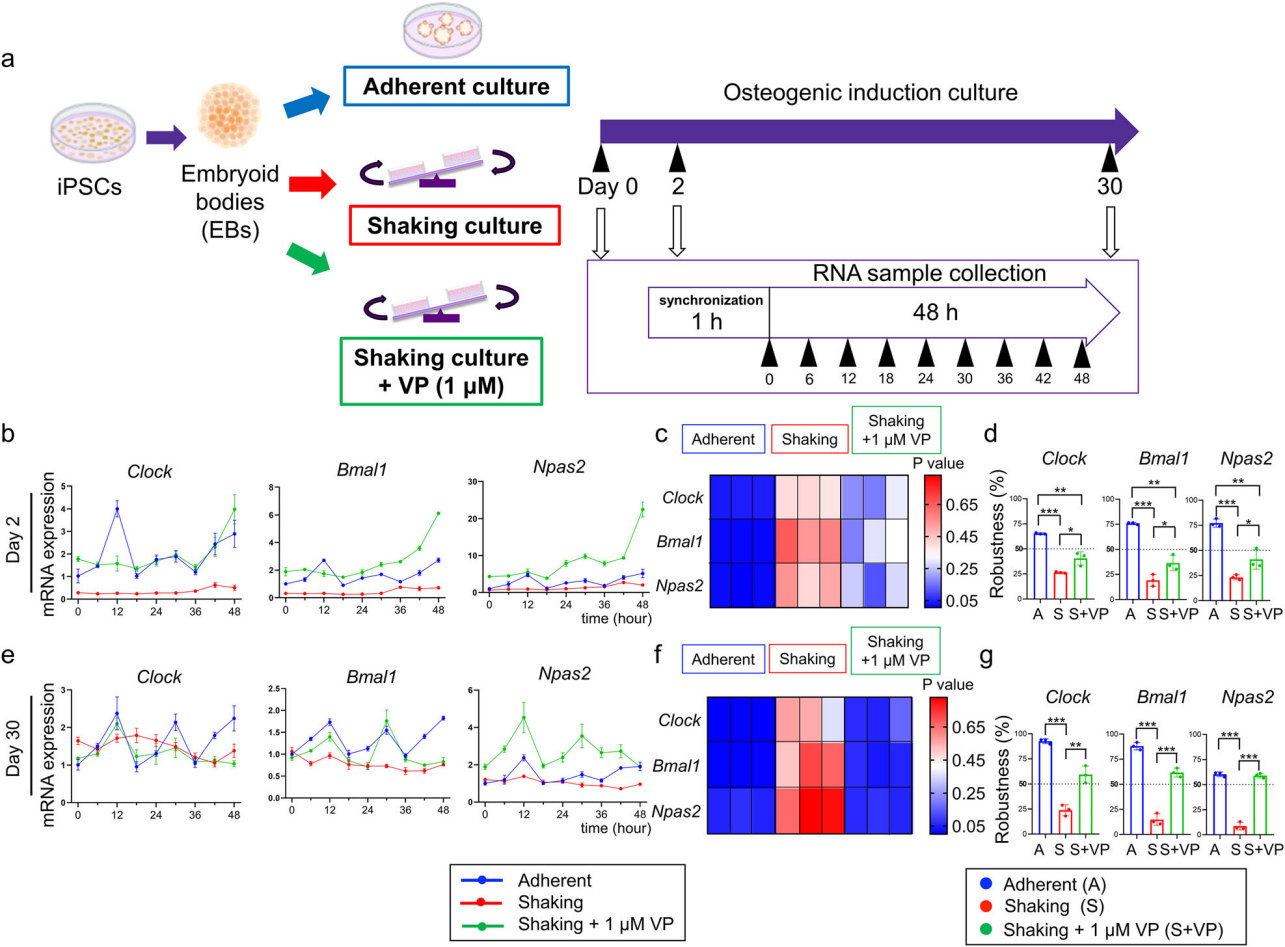
VP treatment restores the rhythmic expression of clock genes in the shaking culture. **a** Schematic illustration of the time course for measuring circadian rhythms. Real-time RT-PCR analysis of *Clock*, *Bmal1*, and *Npas2* mRNA levels at specified time points in iPSC-EBs on days 2 (**b**) and 30 (**e**). *P* values were obtained using Cosinor software by comparing the real-time RT-PCR curve to the standard cosine curve on days 2 (**c**) and 30 (**f**). Three grids in each group represent triplicate experiments. Robustness values were obtained on days 2 (**d**) and 30 (**g**) using Cosinor software. *P < 0.05, **P < 0.01, ***P < 0.001 (one-way ANOVA and Tukey’s test; *n* = 3).

We investigated whether the inhibition of the YAP-TEAD interaction could restore the rhythmic expression of clock genes in shaking cultures. VP was added to the shaking culture during osteogenic induction. *Clock, Bmal1*, and *Npas2* expressions were analyzed in the culture groups with or without VP treatment (Fig. 5a). VP treatment resulted in a decreasing *P* value in the shaking group on day 2 of osteogenic differentiation, although this value did not fall below 0.05 (Fig. 5b, c). VP treatment significantly increased the robustness value in the shaking culture group compared with that in the untreated cells, although it remained lower than that in the adherent group (Fig. 5d). After 30 d of VP treatment, the shaking group exhibited clear rhythmic clock gene expression (*P* < 0.05 and increased robustness values; Fig. 5e–g). These results suggest that the inhibition of YAP-TEAD-mediated transcription restores the attenuated rhythmic expression of clock genes in the shaking culture.

### Inhibition of YAP-TEAD interaction promoted osteogenic differentiation of iPSCs in the shaking culture

**Fig. 6:**
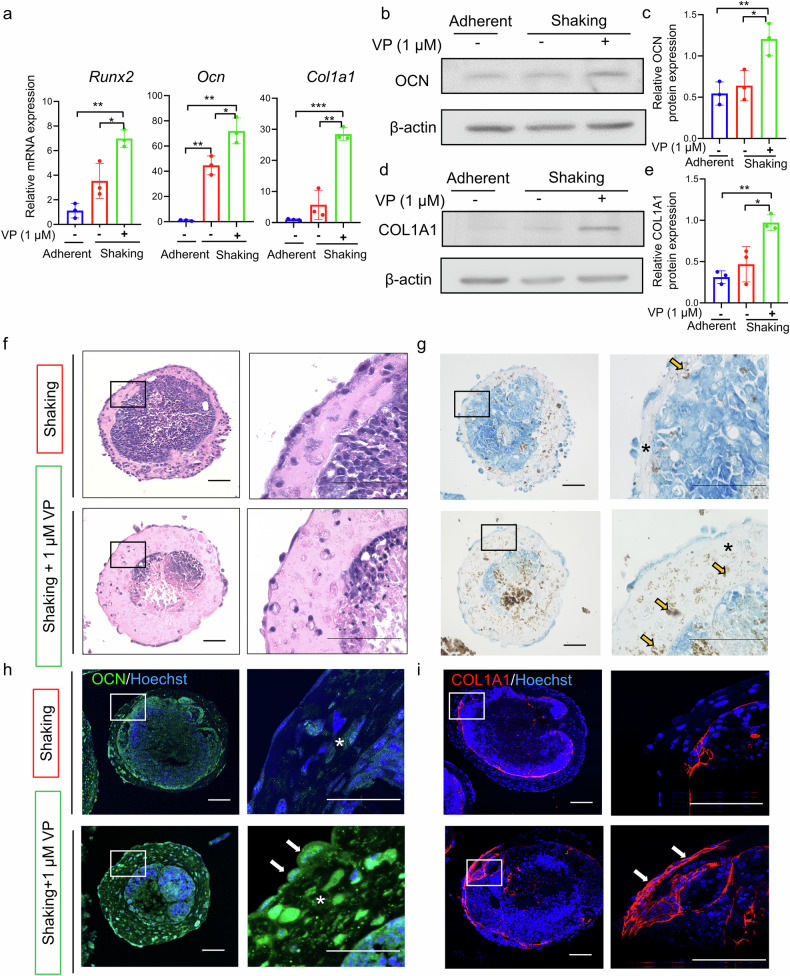
YAP-TEAD interaction inhibitor enhances the osteogenic differentiation of iPSC-EBs. **a** Real-time RT-PCR analysis of the mRNA levels of the osteogenic genes *Runx2*, *Col1a1*, and *Ocn* after 30 d of osteogenic induction. **b** Western blot analysis of the osteogenic marker OCN. **c** Quantitative analysis of OCN expression using ImageJ software. Expression levels were normalized to that of β-actin. **d** Western blot analysis of the osteogenic marker COL1A1. **e** Quantitative analysis of COL1A1 expression using ImageJ software. Expression levels were normalized to that of β-actin. *P < 0.05, **P < 0.01, ***P < 0.001 (one-way ANOVA and Tukey’s test; *n* = 3). **f**, **g** H&E and von Kossa staining of osteogenically induced iPSC-EB after 30 d of culture. Asterisks indicate osseous-like tissue regions and yellow arrows highlight calcified nodules. **h**, **i** Immunofluorescence staining analysis of OCN and COL1A1 in iPSC-EBs. Asterisks indicate osseous-like tissue regions, and white arrows mark positive cell staining for type I collagen and osteocalcin. Scale bars, 50 μm.

To investigate the effect of TEAD-mediated rhythm regulation on osteogenesis, VP was added to the shaking culture during osteogenic induction. The VP-treated shaking group showed significantly upregulated osteogenic marker genes Runt-related transcription factor 2 (*Runx2*), osteocalcin (*Ocn*), and type 1 collagen (*Col1a1*) (Fig. 6a). Western blot analysis confirmed the increased levels of OCN and COL1A1 in the VP-treated group (Fig. 6b–e).

The effect of VP-induced rhythmic restoration on the osteogenic differentiation of iPSCs in shaking cultures was evaluated histologically. Hematoxylin and eosin (H&E) staining revealed that osteogenically induced iPSCs had an inner core of disorganized cell clusters surrounded by layers of bone-like structures, with cell nuclei embedded within an abundant bone-like extracellular matrix. On the outer surface, the cells are arranged in multiple layers or monolayers. The bone-like structural layers were thicker in the VP-treated group (Fig. 6f). von Kossa staining confirmed abundant calcification in the peripheral bone-like matrix with thicker layers and more calcified nodules in the VP-treated group, suggesting increased osteogenic differentiation (Fig. 6g).

We examined the distribution of osteogenic markers via immunofluorescence staining. OCN, a bone matrix protein secreted by osteoblasts, is expressed throughout the bone-like matrix and peripheral cell layers. Compared with the sporadic distribution in the untreated group, the VP-treated group exhibited stronger and increased OCN expression (Fig. 6h). COL1A1 expression was mainly localized in the peripheral cell layer and in some cells within the bone-like areas. Similarly, COL1A1 expression was more pronounced and widespread in the VP-treated group (Fig. 6i).

## Discussion

In mechanobiology, mechanical factors have become integral to culture models used in tissue engineering. In particular, shaking culture modifies cellular morphology and intercellular contacts while delivering sustained hydrodynamic shear and tension to cells [27, 28]. In our previous study, iPSC-EBs in shaking cultures differentiated into well-organized 3D bone- or cartilage-like structures, demonstrating that mechanical forces can guide stem cell lineage specification [13, 28]. Elucidating how mechanical stimuli affect circadian rhythms in tissue engineering can contribute to the improvement of tissue engineering approaches and the development of novel therapeutic strategies.

In this study, iPSC-EBs maintained robust circadian oscillations under adherent conditions but exhibited attenuated rhythms in shaking cultures. These results aligned with recent findings that biomechanical forces differentially affect circadian rhythms across cell types. Fibroblasts exhibit more robust circadian oscillations on stiff substrates, whereas epithelial cells exhibit stronger rhythmicity on soft matrices, possibly because of differential regulation by myocardin-related transcription factors [29]. NIH3T3 fibroblasts seeded in larger areas with greater traction forces exhibited reduced circadian oscillations of Rev-erbα, and this effect was independent of cell–cell adhesions and negatively correlated with nuclear YAP [23]. Overall, mechanical stimuli are important in intracellular circadian rhythm regulation and ultimately affect the systemic circadian clock.

We elucidated the molecular mechanisms underlying circadian rhythm attenuation in iPSC-EBs in shaking cultures. KEGG enrichment analysis revealed significant suppression of ECM-receptor interaction and focal adhesion pathways in shaking cultures, likely reflecting reduced cell–cell contacts. Focal adhesion components were downregulated in 3D-cultured iPSCs compared with 2D cultures [30]. Notably, the Hippo signaling pathway was enriched (Fig. 2d). Inhibition of the Hippo signaling pathway dephosphorylates YAP, enabling its nuclear translocation and function as a transcriptional coactivator with TEAD [31]. YAP lacks a DNA-binding domain and relies on TEAD for transcriptional regulation; when the Hippo pathway is activated, YAP is phosphorylated, preventing its nuclear translocation and impairing TEAD target gene regulation [32, 33].

Significant enrichment of the TEAD2 motif was identified in the shaking group, reinforcing the role of YAP-TEAD in cellular responses to mechanical stress (Fig. 3b). YAP-TEAD signaling influences various cellular processes [34], and increased TEAD-driven transcription disrupts Rev-erbα circadian regulation [23]. In addition, YAP nuclear translocation can be influenced by cytoskeletal dynamics, which are regulated by the RhoA/ROCK pathway [35]. Excessive mechanical strain can also inhibit circadian rhythms in nucleus pulposus cells via the RhoA/ROCK pathway [36]. These findings suggest that the YAP-TEAD transcriptional cascade is a key mediator of circadian rhythm disruption in shaking cultures.

We examined the YAP-TEAD target genes affecting core circadian genes, focusing on *Fbxl3*, which regulates CRY ubiquitination and degradation [37, 38]. Fbxl3 mRNA and CRY protein expression levels confirmed that TEAD-mediated Fbxl3 upregulation enhanced CRY degradation in the shaking culture. Consequently, the abnormal CRY accumulation attenuated the oscillations of the core clock genes in the shaking group, which was reversed by VP treatment. These results indicate that the shaking culture modulated cellular rhythmicity via the TEAD-FBXL3-CRY axis, clarifying how mechanical environments regulate circadian rhythms.

In the present study, VP treatment effectively promoted the osteogenic differentiation of iPSC-EBs. VP has been shown to inhibit YAP/β-catenin signaling and reduce osteogenesis in tendon stem cells [39]. YAP plays different roles in osteoblast differentiation at various stages of mouse embryonic development [40]. VGLL4, another YAP-TEAD inhibitor, promotes Runx2 activity and osteoblast differentiation in mesenchymal stem cells [41]. YAP can also directly interact with the Runx2 protein through the PY motif in osteoblasts and regulate Runx2 activity [42]. YAP knockout leads to reduced expression of osteogenic genes such as *Runx2* and *Sp7*, resulting in bone loss [40]. Thus, the effects of YAP signaling on osteogenic differentiation are complex, potentially involving both direct regulation and mediation through circadian rhythms.

Previous studies implicated the YAP/TAZ activity in promoting osteogenic differentiation. TAZ has been reported to function as an endogenous coactivator of Runx2 and to play a role in the differentiation process of mesenchymal stem cells (MSCs) into osteoblasts [43]. Additionally, it has been demonstrated that YAP and TAZ cooperatively regulate transcriptional events downstream of Runx2 activation, playing an essential role in osteogenic differentiation of MSC [44]. Our results indicated that YAP/TEAD activation suppressed osteogenesis in the shaking culture. This discrepancy was likely because YAP functions as a transcriptional coactivator in shaking culture to induce the TEAD-Fbxl3-CRY axis rather than cooperating with TAZ directly binding to DNA. Notably, VP did not affect the osteogenic differentiation of iPSC-EBs in adherent cultures (Supplementary Fig. 3) but significantly promoted differentiation in shaking cultures. This may be attributed to circadian rhythms being attenuated in shaking cultures but being well-maintained in adherent cultures. Our findings suggest that VP may be used as a small molecule-based strategy for alleviating osteogenic deficits caused by circadian rhythm disruption.

**Fig. 7:**
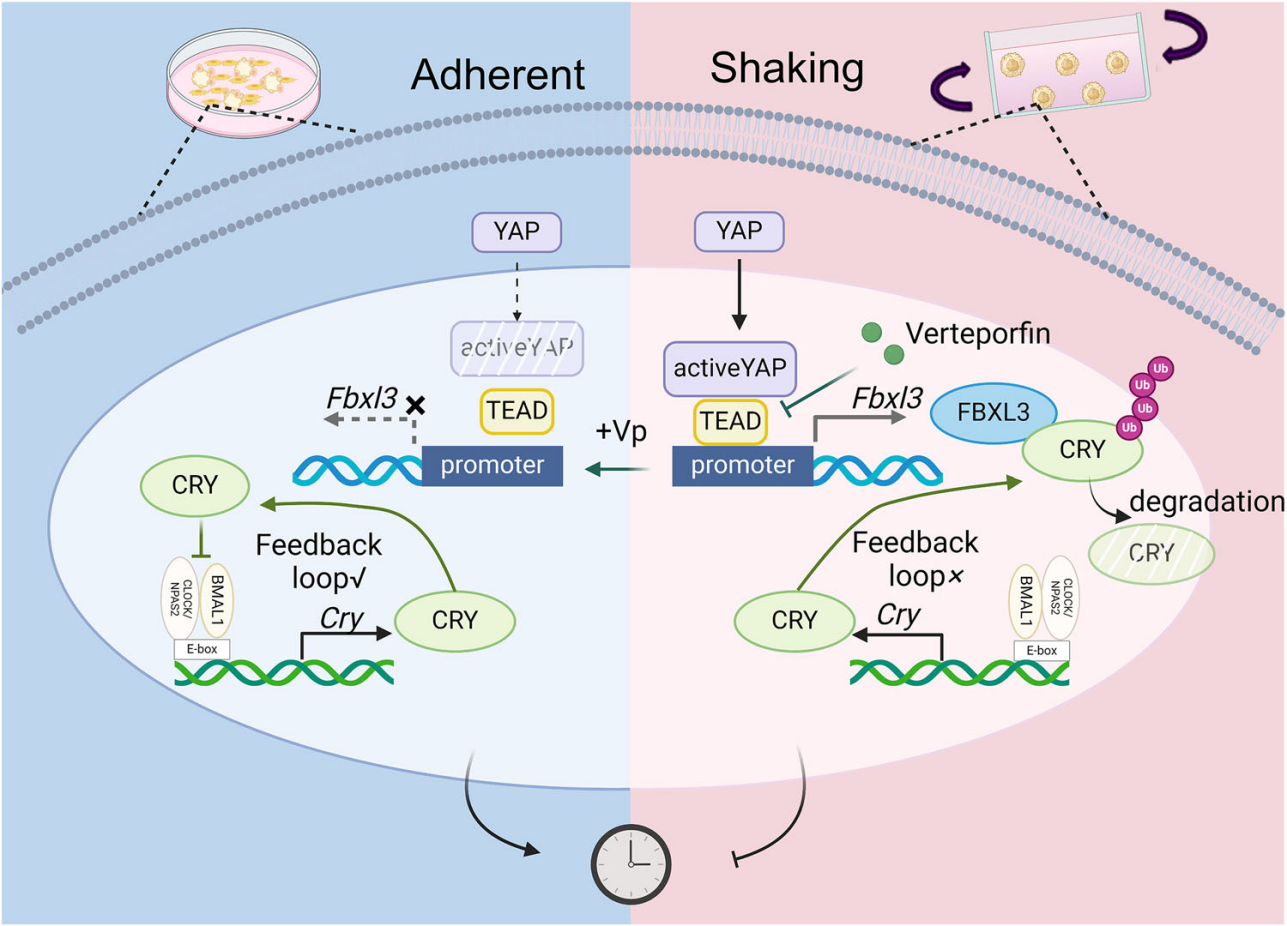
Schematic of the mechanism by which shaking culture attenuates the rhythmic oscillation of clock genes. The mechanical force from the shaking culture activates YAP signaling, promoting Fbxl3 transcription via the TEAD transcription factor. FBXL3 binds to CRY proteins and triggers their ubiquitination and degradation. When abnormally degraded, CRY proteins, which are key regulators of the circadian negative feedback loop, disrupt the loop, leading to the loss of the rhythmic expression of *Clock, Bmal1*, and *Npas2*. This effect is reversed by the addition of verteporfin, which prevents TEAD from initiating *Fbxl3* transcription. Consequently, normally expressed CRY proteins suppress the transcriptional activation of the CLOCK/NPAS2-BMAL1 complex. This complex activates Cry transcription, forming an effective negative feedback loop.

In conclusion, TEAD mediates circadian rhythm attenuation in iPSCs in shaking cultures during osteogenic differentiation through the TEAD-Fbxl3-CRY axis (Fig. 7). This study revealed the relationship between the circadian rhythm, mechanical force, and osteogenic differentiation of iPSCs and provides a theoretical basis for the development of improved methods for iPSC-based bone-organoid fabrication.

## Materials and methods

### Mouse iPSC maintenance on feeder cells

Mouse iPSCs were derived from gingival fibroblasts of C57BL/6J mice, as previously established [45]. iPSCs were maintained on inactivated SNL feeder cells in an ES medium containing Dulbecco’s modified eagle medium with 4.5 g/L glucose without sodium pyruvate (Nacalai Tesque, Kyoto, Japan), 15% fetal bovine serum (FBS) (Biosera, France), 2 mM l-glutamine, 50 µg/mL penicillin (50 U)-streptomycin (Wako Pure Chemical Corporation, Japan), 1 × 10^−^^4 ^M nonessential amino acids, and 1 × 10^−4 ^M 2-mercaptoethanol (Thermo Fisher Scientific, MA, USA). The cells were passaged every 5–6 d.

### Osteogenic induction culture under adherent and shaking conditions

Osteogenic induction of iPSCs was performed as previously described [13]. After embryoid body (EB) formation using low-attachment culture dishes, 1 mM retinoic acid (Wako Pure Chemical Corporation) was added to the ES medium for 2 d [46].

For the adherent culture, the EBs were seeded in 0.1% gelatin-coated six-well plates and the medium was switched to osteogenic induction medium containing alpha minimum essential medium (Nacalai Tesque), 15% FBS (Thermo Fisher Scientific), 0.01 µM dexamethasone, 10 mM β-glycerophosphate, 50 µg/mL ascorbate-2-phosphate (Sigma-Aldrich, MO, USA), and 1% antibiotic-antimycotic solution (Thermo Fisher Scientific).

For the shaking culture, EBs were cultured in low-attachment culture flasks (Thermo Fisher Scientific) containing an osteogenic induction medium. The flasks were then placed on a seesaw shaker with a constant frequency of 0.3 Hz. In both groups, the osteogenic medium was replaced every 2 d.

### Real-time reverse transcription-PCR (RT-PCR) analysis

To examine the clock gene expression profile after 0, 2, 20, and 30 d of osteogenic induction, 0.1 mM dexamethasone was applied to the cells for synchronization 2 h before total RNA extraction, as described previously [47] (Fig. 1a). Real-time RT-PCR was performed using TaqMan™ Fast Advanced Master Mix (Thermo Fisher Scientific) and TaqMan probes (*Clock* [Mm00455950_m1], *Bmal1* [Mm00500223_m1] and *Npas2* [Mm01239312_m1]). Osteogenic gene expression was analyzed using Thunderbird SYBR qPCR Mix (Toyobo, Osaka, Japan). The primer sequences are listed in Supplementary Table 2.

To quantify rhythmic oscillations, a 48-hour cycle of gene expression was analyzed using Cosinor software (https://www.circadian.org/softwar.html). Cosinor simulates a cosine curve based on input data and provides several parameters for evaluating whether the dataset exhibits rhythmic oscillations [48]. The *P*-value was obtained by comparing the RT-PCR curve with the standard cosine curve. The robustness value indicates the regularity of the oscillations, with a larger value suggesting a more regular oscillation that signifies a better rhythmic pattern. *P* < 0.05 and robustness value > 50% indicate a rhythmic oscillation of gene expression.

### Western blotting

Total protein was extracted using radioimmunoprecipitation Assay buffer (Wako Pure Chemical Corporation) containing protease inhibitors (Cytoskeleton, USA). The proteins were mixed with 4x Laemmli sample buffer (Bio-Rad Laboratories, CA, USA) and loaded onto a sodium dodecyl sulfate-polyacrylamide gel. The separated proteins were transferred onto polyvinylidene fluoride membranes (Bio-Rad Laboratories) and blocked with 5% skim milk for 1 h at room temperature (RT). Primary antibodies were used to detect active YAP, CRY, COL1A1, OCN, and β-actin. Subsequently, the corresponding anti-mouse/rabbit IgG-horseradish peroxidase antibodies (Santa Cruz Biotechnology, TX, USA) were used. The antibodies used in this study are listed in Supplementary Table 3.

### RNA-seq and data analysis

Total RNA was collected from the cultures 2 d after osteogenic induction. The samples were treated with DNase (Thermo Fisher Scientific) to remove DNA. mRNA sequencing was performed on an Illumina 6000 platform (Macrogen, Japan) with a 101 bp read length. All adapters and low-quality reads were removed using FastQC. The R package ‘DESeq2’ was used for analysis, with a threshold of *P* value < 0.05 and fold change > ±1.2 for differentially expressed genes (DEGs). The KEGG pathways of the DEGs were identified using the KEGG Orthology-Based Annotation System.

### ATAC-seq

Cell pellets (1 × 10^5^ cells per vial) were collected from the cultures 2 d after osteogenic induction. ATAC-seq was performed as previously described [49]. SAMtools [50] and deepTools [51] were used to remove low-quality mapped reads and convert BAM files into bigWig files, respectively. Random genomic regions equal in number and size to the ATAC peaks were selected, and their average coverage signals were measured to determine the background value. Peaks were annotated using the annotatePeaks.pl script in the HOMER package.

### ChIP assays

ChIP was performed using a chromatin immunoprecipitation kit (Cell Signaling Technology) following the manufacturer’s instructions. Proteins and DNA were cross-linked, lysed, and enzymatically digested into suitable DNA fragments (150–900 bp). Two percent of the samples were removed as input samples before antibody incubation. Next, the samples were incubated with TEAD antibody (Pan-TEAD, 1 μg; Cell Signaling Technology) and Protein G Agarose Beads sequentially at 4 °C with rotation. The immunoprecipitated complexes were de-crosslinked and purified to obtain the final DNA for qPCR. The primer sequences were designed based on the promoter regions of the ATAC sequences (Supplementary Table 4).

### Histological and immunofluorescence analyses

The paraffin-embedded iPSC-EB sections were deparaffinized and hydrated. For H&E staining, the sections were immersed in hematoxylin for 5 and eosin for 2 min, followed by dehydration. For methylene blue-counterstained von Kossa staining, the sections were treated with a 5% silver nitrate solution (Wako Pure Chemical Corporation) under ultraviolet light for 10 min, followed by 1% methylene blue solution (Muto Chemicals, Tokyo, Japan) for 5 min. For immunofluorescence staining, antigen was retrieved using 10 mM citrate buffer (pH 6.0; Wako Pure Chemical Corporation) for 10 min at 121°C before antibody incubation. The antibodies used are listed in Supplementary Table 3.

### Statistical analysis

Multiple groups were compared using one-way analysis of variance (ANOVA) with Dunnett’s or Tukey’s post-hoc test. Statistical significance was set at *P* < 0.05. Error bars represent standard deviation.

## Supplementary information

Supplementary Figures and Tables with Legends

Original western blots

## Data Availability

The datasets generated and/or analyzed during the current study are available from the corresponding authors on reasonable request. RNA and ATAC sequence data have been deposited in the GEO under accession code GSE291628.
