# Supplementary material for: Shaking culture attenuates circadian rhythms in induced pluripotent stem cells during osteogenic differentiation through the TEAD-Fbxl3-CRY axis
Source: Cell Death Discov. 2025 May 24;11:252. doi: 10.1038/s41420-025-02533-6 (PMC12103599; doi:10.1038/s41420-025-02533-6)
Supplement: Supplementary file 1 — Supplementary Figures and Tables with Legends [file 41420_2025_2533_MOESM1_ESM.docx]

**Supplementary Figures**

**Supplementary Fig. 1:**

**Top 10 motifs enriched in the promoters of the upregulated genes in the adherent culture group**. Related to Fig. 3b.


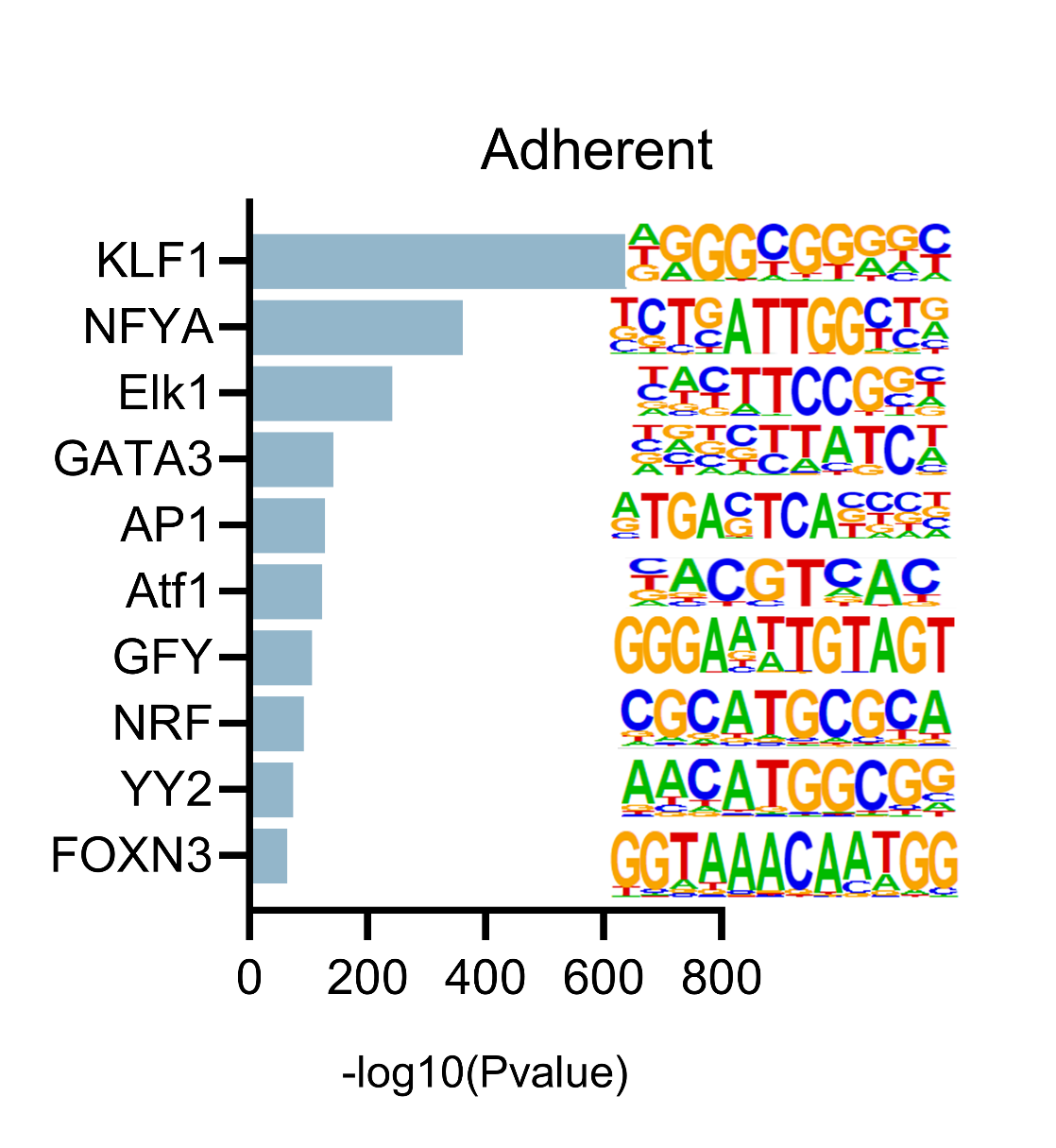


**Supplementary Fig. 2:**

**ChIP assay analysis of the interaction between TEAD and two other candidates***.* Related to Fig. 4a. **a** ChIP-qPCR analysis of the interaction between TEAD and the *Rorb* promoter. **b** ChIP-qPCR analysis of the interaction between TEAD and the *Cry1* promoter. The input represents an aliquot of total DNA. **P*<0.05, ****P*<0.001 *****P*<0.0001 (one-way ANOVA and Tukey’s test; n=3).


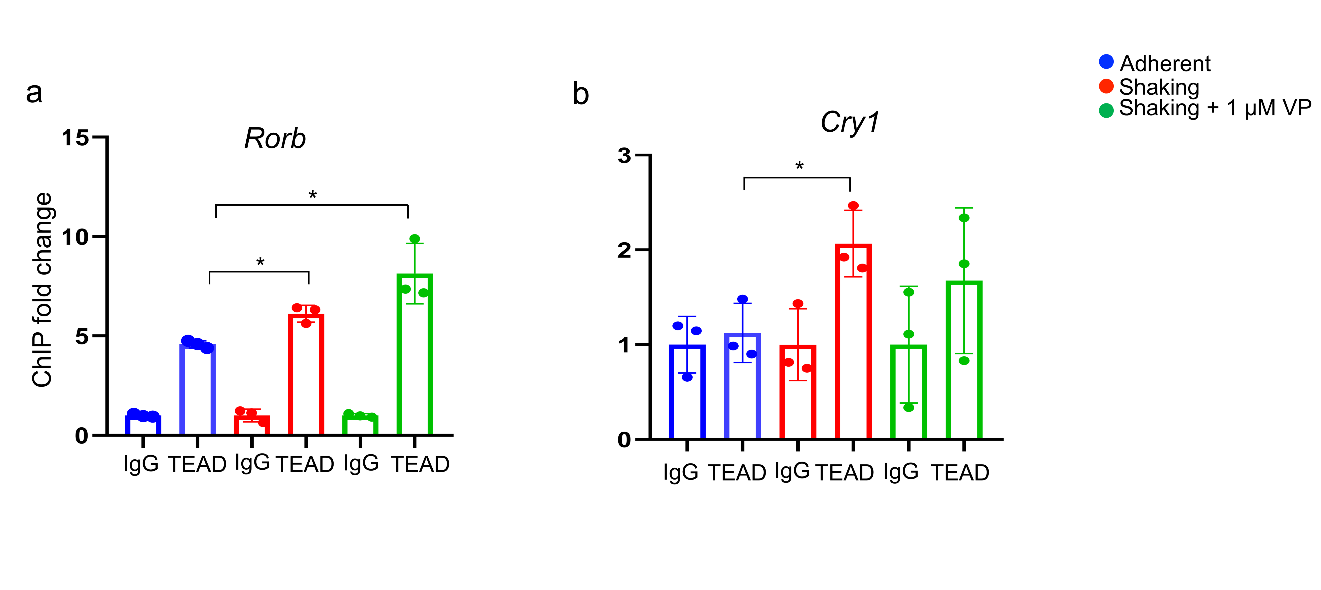


**Supplementary Fig. 3:**

**Effect of verteporfin treatment on the osteogenic differentiation of iPSC-EBs in adherent cultures. a** Real-time RT-PCR analysis of the mRNA levels of the osteogenic genes *Runx2*, *Col1a1*, and *Ocn* after 30 d of osteogenic induction. **b** Methylene blue-counterstained von Kossa staining of iPSC-Ebs cultured under adherent conditions with or without verteporfin treatment. ***P*<0.01 (one-way ANOVA and Tukey’s test; n=3).


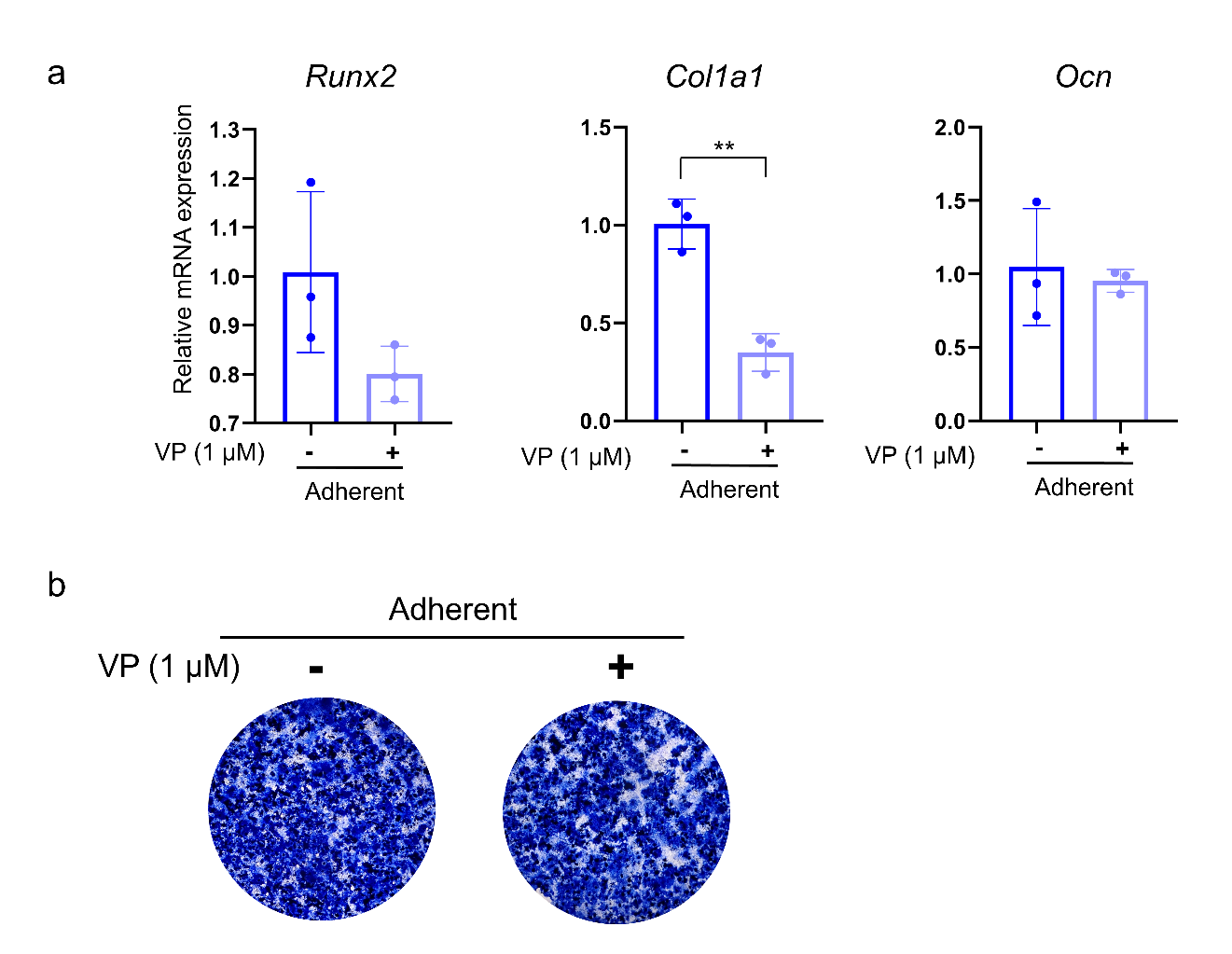


**Supplementary tables**

**Supplementary Table 1: Upregulated clock genes in RNA-seq**

| Rank | Gene | Gene_ID | FC |
| --- | --- | --- | --- |
| 1 | *Prkag3* | 241113 | 13.7396 |
| 2 | *Npas2* | 18143 | 6.6182 |
| 3 | *Rorb* | 225998 | 3.8099 |
| 4 | *Per3* | 18628 | 1.7911 |
| 5 | *Prkab1* | 19079 | 1.6757 |
| 6 | *Cry1* | 12952 | 1.5826 |
| 7 | *Fbxl3* | 50789 | 1.3495 |
| 8 | *Prkaa2* | 108079 | 1.2836 |

**Supplementary Table 2: Primer list for quantitative real-time RT-PCR.**

| **Target gene** | **Forward primer (5′-3′)** | **Reverse primer (5′-3′)** |
| --- | --- | --- |
| *Gapdh* | TGCACCACCAACTGCTTAG | GGATGCAGGGATGATGTTC |
| *Runx2* | CGGGCTACCTGCCATCAC | GGCCAGAGGCAGAAGTCAGA |
| *Col1a1* | TGTCCCAACCCCCAAAGAC | CCCTCGACTCCTACATCTTCTGA |
| *Ocn* | CCGGGAGCAGTGTGAGCTTA | AGGCGGTCTTCAAGCCATACT |
| *Fbxl3* | ACCACCTACAGTATGTCAGC | AGTCGAGATAAGTCCGAGAG |

**Supplementary Table 3: Antibody lists for immunostaining and western blotting**

| **Antibody** | **Cat No.** | **Manufacturer** | **immunostaining** Dilution, temperature, duration | **western blotting** Dilution, temperature, duration |
| --- | --- | --- | --- | --- |
| OCN | sc390877 | Santa Cruz | 1:80, 4℃, 8 h | 1:100, 4℃, 8 h |
| COL1A1 | NBP1-30054 | Novus Biologicals | 1:100, 4℃, 8 h | 1:1000, 4℃, 8 h |
| Active-YAP1 | ab205270 | Abcam | - | 1:800, 4℃, 8 h |
| CRY | 13997-1-AP | proteintech | - | 1:800, 4℃, 8 h |
| β-actin | 3700 | Cell signaling | - | 1:2000, 4℃, 8 h |
| Hoechst | H3569 | Invitrogen | 1:1000, 25℃, 8min | - |
| anti-rabbit IgG-HRP | 7074 | Cell signaling | - | 1:4000-1:2000, 25℃, 1 h |
| anti-mouse IgG-HRP | sc-516102 | Santa Cruz | - | 1:4000-1:1000,  25℃, 1 h |
| anti-mouse IgG-Alexa 488 | Ab150113 | Abcam | 1:500, 25℃, 1 h | - |
| anti-rabbit IgG-Alexa 647 | ab150075 | Abcam | 1:500, 25℃, 1 h | - |

**Supplementary Table 4: Primer list for ChIP (ATAC sequences)**

| **Target gene** | **Forward primer (5′-3′)** | **Reverse primer (5′-3′)** |
| --- | --- | --- |
| *Rorb* | TCTTTCCCAGAACACCAATTACTT | CTTAACCATTGAGCCACCTATACA |
| *Fbxl3* | AGATTGTGAGAGTCAAAGGAACAG | ATTGGTGTTCTTGTCATTTGGGTA |
| *Cry1* | CTGATTCAGTTGAGTTGCAGTTTT | CAAGGAGAGAAAGCAAAGGTGTTA |
