# Supplementary material for: Shaking culture attenuates circadian rhythms in induced pluripotent stem cells during osteogenic differentiation through the TEAD-Fbxl3-CRY axis
Source: Cell Death Discov. 2025 May 24;11:252. doi: 10.1038/s41420-025-02533-6 (PMC12103599; doi:10.1038/s41420-025-02533-6)
Supplement: Supplementary file 2 — Original western blots [file 41420_2025_2533_MOESM2_ESM.docx]

**Western blots in Fig. 3e and ChIP-qPCR assay in Fig. 4b**


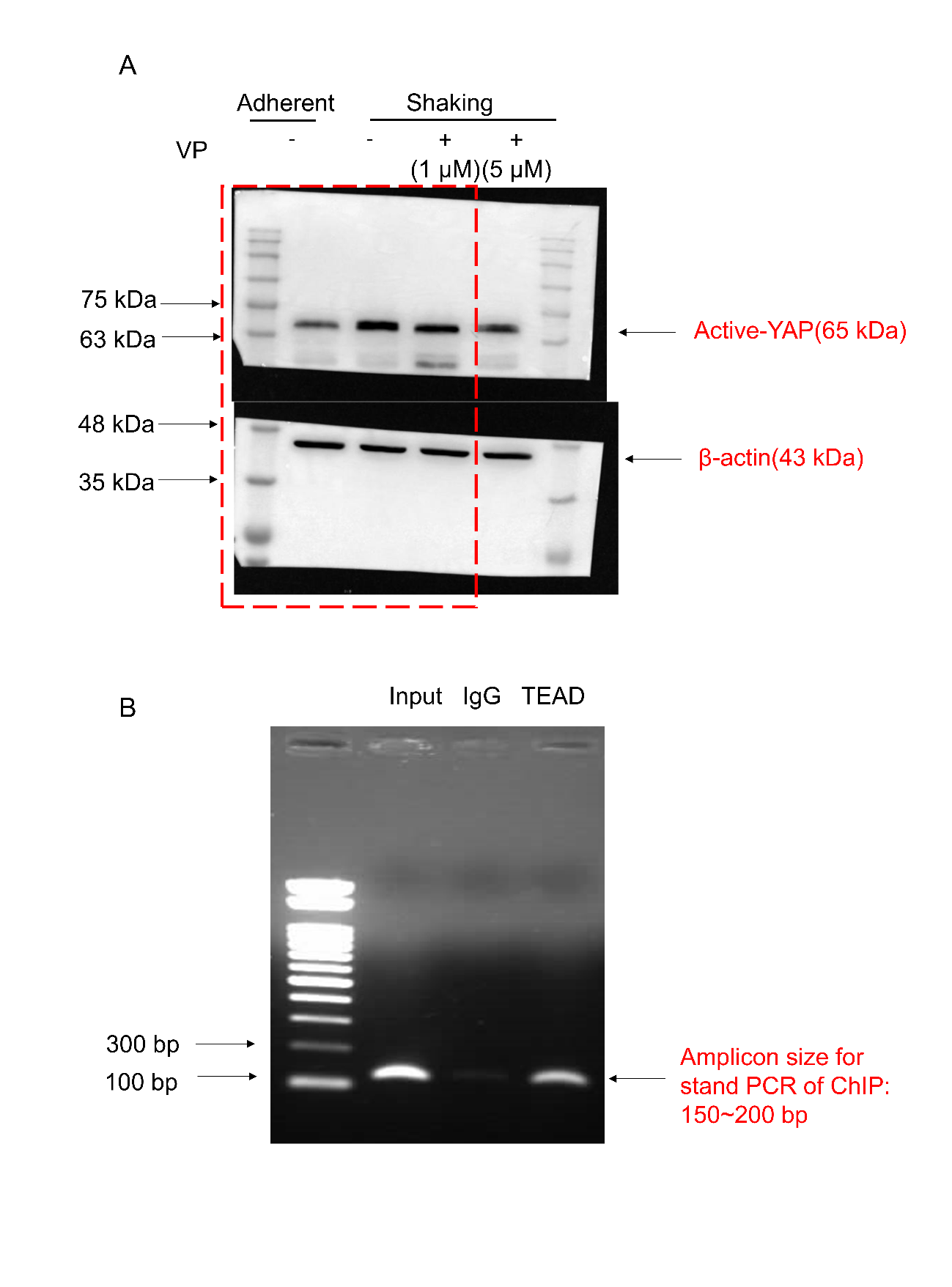


(**A**) Raw data of active YAP proteins using western blotting analysis in Fig. 3e

(**B**) Raw data of stand PCR of ChIP assay in Fig. 4b.

**Western blots in Fig. 4e and 4g**


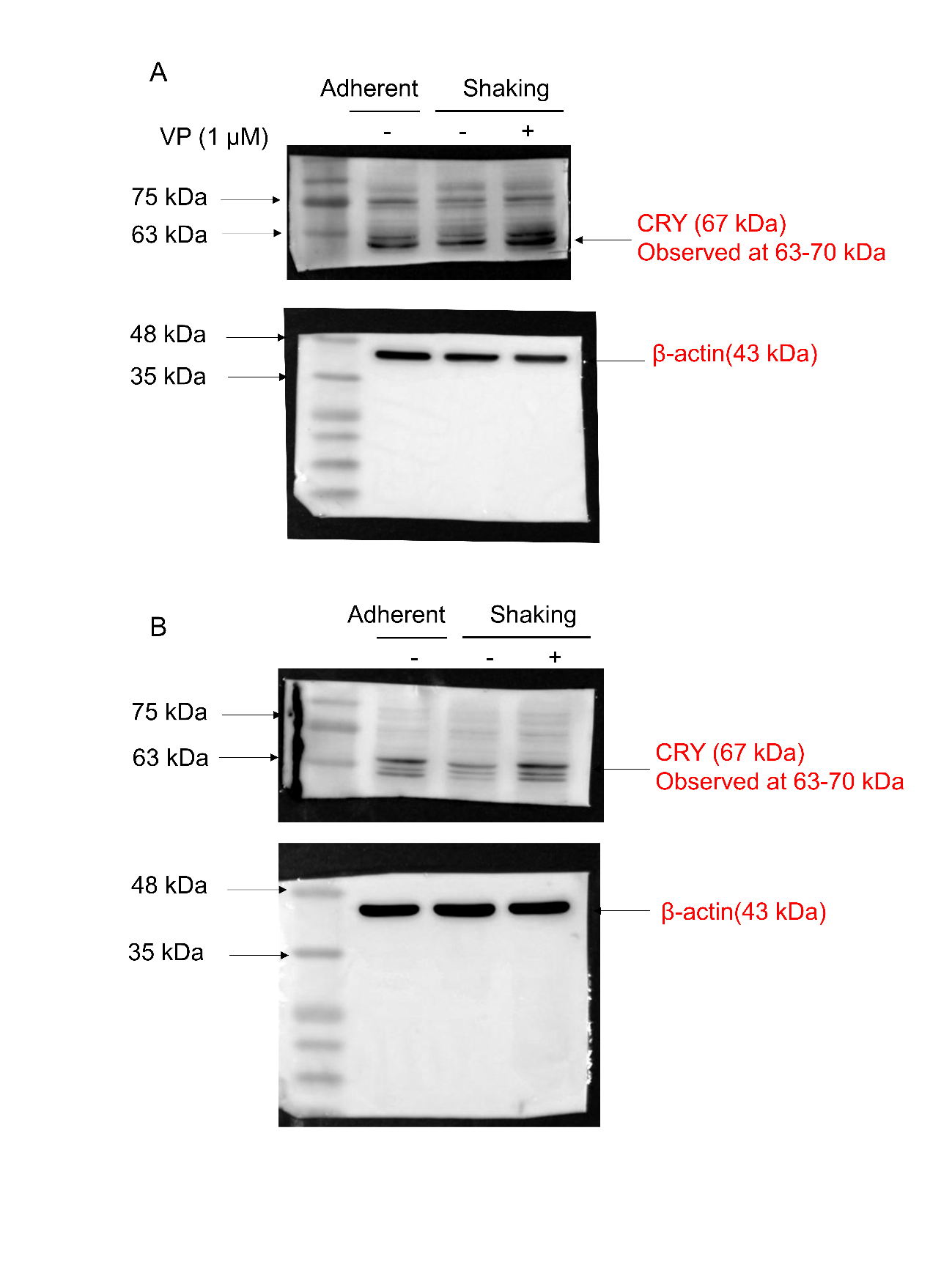


**(A)** Raw data of the expression of CRY proteins, as demonstrated in Fig. 4e.

**(B)** Raw data of the expression of CRY proteins, as demonstrated in Fig. 4g.

**Western blots in Fig. 6b and 6d**


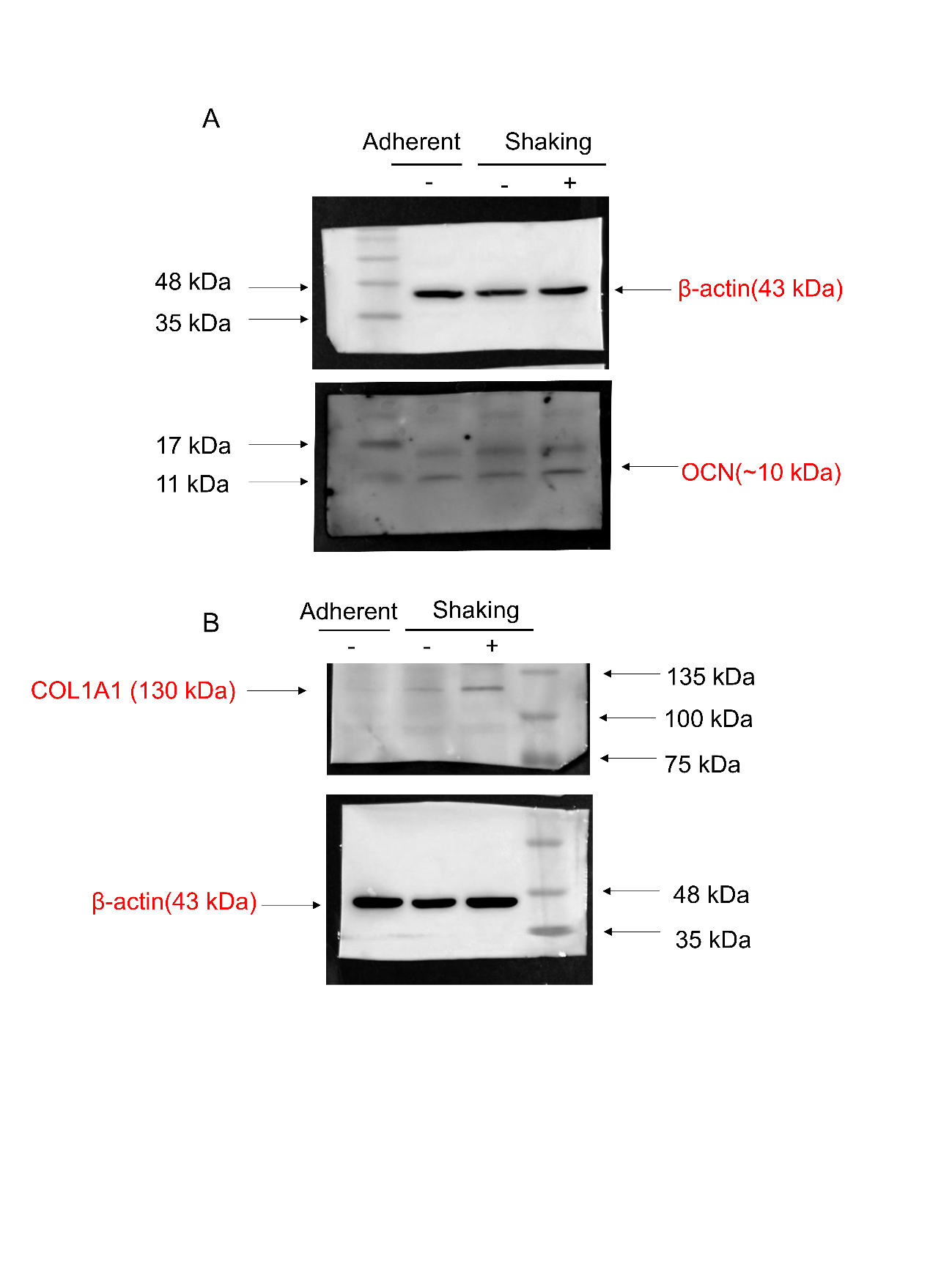


**(A)** Raw data of the expression of osteocalcin proteins, as demonstrated in Fig. 6b.

**(B)** Raw data of the expression of type I collagen proteins, as demonstrated in Fig. 6d.
